# Supplementary material for: Significantly higher atherosclerosis risks in patients with obstructive sleep apnea and non-alcoholic fatty liver disease
Source: PLoS One. 2021 Jun 30;16(6):e0253298. doi: 10.1371/journal.pone.0253298 (PMC8244858; doi:10.1371/journal.pone.0253298)
Supplement: Attachment — Submitted filename: Response Letter for Reviewers Final.docx [file pone.0253298.s002.docx]

14^th^ April 2021

Editor

PLOS ONE

Dear Sir,

Thank you for your response and providing us the opportunity to revise the report accordingly. I hereby provide the details of the amendments as required by the Editors.

a) If there are ethical or legal restrictions on sharing a de-identified data set, please explain them in detail (e.g., data contain potentially identifying or sensitive patient information) and who has imposed them (e.g., an ethics committee). Please also provide contact information for a data access committee, ethics committee, or other institutional body to which data requests may be sent.

*I have clarified the issue of data availability. We should be able to provide the data set as requested.*

b) If there are no restrictions, please upload the minimal anonymized data set necessary to replicate your study findings as either Supporting Information files or to a stable, public repository and provide us with the relevant URLs, DOIs, or accession numbers.

*The anonymized data set in the SPSS for has been uploaded as Supporting Information Files*

We would like to thank both Reviewers for their constructive comments and suggestions. We hereby provide the responses to the questions and comments.

Reviewer #1:

Degree of steatosis is not the same as stage/grade of NAFLD. Grade is determined by degree of inflammation (NAS score) and stage by fibrosis on biopsy (gold standard). If biopsy is not available severity can be assessed by specialized non invasive markers and scores like Fibroscan, NALFD fibrosis score etc. Hence in this paper the term grade/stage of NAFLD should be replaced by degree of steatosis.

*Thank you for the clarification. This has been amended accordingly throughout the manuscript. Stages of NAFLD has been replaced as grades of steatosis Grade 0-4.*

It would be useful if the authors had any such surrogate markers of NAFLD severity and study the relationship of NAFLD severity with atherosclerosis markers.

*Thank you for the suggestion. Unfortunately we do not have access to Fibroscan. This is a very good suggestion for us to continue with the study at a later opportunity. We have included this in the limitation as well.*

What is the significance of table 5? It would seem that the authors are saying that in patients who do not have NAFLD, CIMT is not related to any metabolic or cardiovascular factor which does not seem to be true clinically. I think there are too few patients in Non NAFLD group to such correlation analysis in this subgroup.

*The reviewer has highlighted a valid point. Table 5 has been removed as suggested. Table 6 has been adjusted to Table 5 accordingly.*

Table 6 and table 7 are almost duplicate. One of them can be removed.

*Thank you for highlighting that. We have removed Table 7. Table 8 has been adjusted to table 6 accordingly.*

How many parameters were entered into the multivariate analysis model? With only 20 patients without NAFLD in the study, doing a multivariate analysis with many dependent variables will severely limit the power of the calculations.

*Thank you for the comments. 10 parameters were included in the multivariate analysis as described in table 7 previously. We have acknowledged the small number of n=20 in the Non-NAFLD group in the result section.*

*This sentence has been added : “All the variables in Table 5 were included in this analysis. Notably, the small number in the non-NAFLD group limit the statistical power. Nevertheless, two factors….”*

Which parameters were studied in the multivariate analysis? It is surprising to note that BMI/obesity did not come out as independent predictors of NAFLD in this study. were these parameters included in the multivariate analysis?

*Only the significant parameters were included in the multivariate analysis ie SBP, DBP, BMI, WC, ALP, ALT, AST, GGT, ICAM-1, Lp(a).*

*Yes, BMI was included in the analysis, however it was not significant.*

"Inversely it could, therefore, be inferred that these OSA patients who had NAFLD had a 95.6% possibility of having elevated atherosclerosis risk as suggested by elevated levels of ICAM-1 and Lp(a)."

I don’t think this is a correct conclusion. AUROC of 95.6% for ICAM and LPa simply suggests that a predictive score using these 2 parameters together has 95.6% accuracy in predicting presence of NAFLD. To assess the probability of atherosclerotic risk in NAFLD one would have to calculate how many of NAFLD patients had elevated levels of one or more markers of atherosclerosis using acceptable cut-offs for each such marker and then calculate the atherosclerotic risk associated with each of those markers.

*Thank you for the comment. The statement has been amended to include your comment.*

*"It could, therefore, be inferred that OSA patients who had elevated levels of ICAM-1 and Lp(a) are highly likely to have NAFLD.”*

"However, there is paucity of data on the cumulative atherosclerotic risk of patients with co-existing OSA
and NAFLD; and to the best of our knowledge this would be the first study utilizing CIMT and the inflammatory markers to demonstrate this."

This is a misleading conclusion. Please see the point above.

*Thank you for the comment. We have removed the statement of “……and to the best of our knowledge this would be the first study utilizing CIMT and the inflammatory markers to demonstrate this."*

"We concur with the findings by Petta et al, which showed that OSA was independently associated with subclinical carotid atherosclerosis as evaluated by CIMT in patients with NAFLD".

The authors have not presented any such data showing association of CIMT with OSA, since there was no non OSA control group in this study to compare CIMT values with.

*Thank you for the comment. We have revised the statement to state the following:*

*Petta et al, showed that in a group of patients with NAFLD who subsequently underwent cardiorespiratory polygraphy to detect OSA, the prevalence of significant thickening of CIMT was higher in patients at high risk compared to those at low risk for OSA (51.2% vs 24.3%, p = 0.006).*

"Furthermore, our data demonstrated a significant rise in ICAM-1 levels with severity of OSA as categorized by
increasing AHI, from mild 259.16 ± 57.40 ng/mL, moderate 299.65 ± 90.41 ng/mL and severe 370.63 ± 57.71 ng/mL, p < 0.001".

This data has not been presented anywhere in the results section.

*Thank you for the comment. You are absolutely right, we have not presented this data in the result section. We have removed this statement in the discussion section.
Reference no 35 has been removed. Subsequent references have been adjusted accordingly.*

The discussion is too long with many repetitions and can be condensed.

*Thank you for the comment. The discussion has been refined to avoid repetitions without losing the relevant findings of the study.*

Reviewer #2: This was an interesting study that examined potential links between OSA, liver disease and CV risks. The authors studied 110 patients with suspected OSA. Authors found that OSA severity was significantly associated with liver disease. CV risk (CIMT) was elevated in liver patients. I had the following questions/comments:

1. In the discussion, other limitations that should be mentioned include: relatively small numbers, single centre, cross sectional nature

*Thank you for the comment. This has been added in the limitation. . “The single center data collection, the cross-sectional design and the relatively small number of subjects are areas of improvement for future studies.”*

2. I wasn’t sure if these were consecutive patients? if not, how were they selected?

*Thank you for the question. Patients were recruited based on convenient sampling. This has been added in the method section.” We screened 110 subjects* ***consecutively,*** *between the ages of 18 to 65, who were diagnosed with OSA….*

3. When was the blood taken in relation to PSG (i.e. time frame)?

*Thank you for the question. PSG was done at a much earlier date just to establish the diagnosis of OSA.*

4. I assume none of these patients were treated?

*Thank you for the question. Upon recruitment, the patients have not initiated treatment yet.*

5. Was there an analysis of desaturation indices (e.g. 4% ODI, mean saturation, % time below 90% etc). I would suspect these might be highly related to liver disease?

*Thank you for the question. We understand the relevance. Unfortunately, these were not available in our analysis.*

6. I wasn’t sure why ICAM specifically was the inflammatory marker selected?

*Thank you for the question. We acknowledge the many surrogate markers for cardiovascular disease including ICAM-1, which is an intercellular adhesion molecule that are expressed on the surface of vascular endothelial cells. It is a valid and recognised research tool, serving as one of the many molecular markers for atherosclerosis and the development of coronary heart disease.*

7. Mean BMI, AHI, saturation, etc should be added to Table 1. Did any patients have obesity hypoventilation syndrome? This might have increased right sided pressures and lead to more liver swelling?

*Thank you for the suggestion. Mean BMI and AHI has been added in Table 1. But we were unable to include the saturation indices. The study also did not look into obesity hypoventilation syndrome and its associated complications. This would however be an interesting variable for future studies in the area.*

8. There should be more description of the ultrasound methods to detect liver disease. How validated are they in the context of substantial obesity +/- high right sided pressures?

*Thank you for the suggestion. We have added the this statement. “Evidence of NAFLD was confirmed with radiological technique of liver-kidney contrast on degree of stetatosis and further divided into four grades, severe (NAFLD-3), moderate (NAFLD-2), mild (NAFLD-1) and normal (non-NAFLD)…..” on Page 9. The examinations were performed by 2 independent, trained radiologists based according to universally accepted criteria (Joy, D., Thava, V. R., & Scott, B. B. (2003). Diagnosis of fatty liver disease: is biopsy necessary? Eur J Gastroenterol Hepatol, 15(5), 539-543. doi:10.1097/01.meg.0000059112.41030.2e)*

9. In the last paragraph of page 8, do you mean carotid ultrasound or abdominal?

*Thank you for highlighting this. We have added – “For the CIMT measurements subjects were also scanned in the supine position by two independent radiologists utilizing the same equipment. The distance between the 2 lines gave a reliable index of the thickness of the carotid intimal-medial complex. All measurements were made at the time of the scan on frozen images of longitudinal scans by using the machine’s electronic caliper. Carotid segments for far (posterior) walls of each common carotid artery at a distance of 1cm from the bulb will be examined. The average of right and left CIMT were calculated and were recorded into millimeters (mm)….”*

10. Was there an analysis of BMI, AHI as continuous variables? It was difficult to understand if Tables 7 and 8 reflected multivariate models? This should be made clearer.

*Thank you for the comment. There were analysis of BMI and AHI as continuous variables and we have added the mean±SD in Table 1. We have removed Tables 5 and 7 as suggested by Reviewer 1. Table 8 has been adjusted to Table 6. It is a multivariate analysis.*

11. The CI around the OR for severe OSA is very wide-- these should be added to the abstract so readers understand the instability of these measures.

*Thank you for the suggestion. This has been added in the abstract.*

12. Was there a multivariate analysis looking at determinants of CIMT? I think you need this to determine whether liver disease is an independent risk factor.

*Thank you for the suggestion. This was not assessed in this paper but worthwhile addressing in future studies.*

Thank you again for your suggestions. We look forward to your positive review.

Sincerely yours,

**Corresponding author:**

**Prof Dr Rohana Abdul Ghani**

Consultant Endocrinologist
Endocrine Unit

Department of Internal Medicine

Faculty of Medicine

Universiti Teknologi MARA (UiTM),

Selangor, Malaysia.
