# Supplementary material for: Significantly higher atherosclerosis risks in patients with obstructive sleep apnea and non-alcoholic fatty liver disease
Source: PLoS One. 2021 Jun 30;16(6):e0253298. doi: 10.1371/journal.pone.0253298 (PMC8244858; doi:10.1371/journal.pone.0253298)
Supplement: Attachment — Submitted filename: Response Letter for Reviewers 2.docx [file pone.0253298.s003.docx]

4^th^ May 2021

Editor

PLOS ONE

**Significantly Higher Atherosclerosis Risks in Patients with Obstructive Sleep Apnoea and Non Alcoholic Fatty Liver Disease** **[PONE-D-21-03573R1]**

Dear Sir,

Thank you again for your kind responses and effort towards this manuscript. I hereby provide the details of the amendments as required by the Editors and Reviewer 2.

4. Have the authors made all data underlying the findings in their manuscript fully available?

The PLOS Data policy requires authors to make all data underlying the findings described in their manuscript fully available without restriction, with rare exception (please refer to the Data Availability Statement in the manuscript PDF file). The data should be provided as part of the manuscript or its supporting information, or deposited to a public repository. For example, in addition to summary statistics, the data points behind means, medians and variance measures should be available. If there are restrictions on publicly sharing data—e.g. participant privacy or use of data from a third party—those must be specified.

Reviewer #1: Yes

Reviewer #2: No

Author response: The raw data has been uploaded as supporting document.

5. Is the manuscript presented in an intelligible fashion and written in standard English?

PLOS ONE does not copyedit accepted manuscripts, so the language in submitted articles must be clear, correct, and unambiguous. Any typographical or grammatical errors should be corrected at revision, so please note any specific errors here.

Reviewer #1: Yes

Reviewer #2: No

Author response: The manuscript has been checked, corrected and revised thoroughly.

Reviewer 2:

The authors have addressed the majority of my comments and the paper improved. I just had a few more:

1. I wasn’t sure why desaturation parameters were not available? Isn’t this part of the PSG report (event indices such as mean saturation, % below 90% would have been useful).

Thank you for the question. I apologize for the ambiguous response previously.

The data is available. However, in relation to the current data presented in this manuscript, the PSG served only as a diagnostic tool to identify patients with OSA and subsequent categorization on severity based on the AHI. Further discussion on OSA would make the manuscript too lengthy and subsequently exceed the stipulated maximum word count.

However, we do acknowledge the relevance of the event indices that you have mentioned above and we enthuse over a future report to determine the associations between those indices and the studied atherosclerosis risks.

2. It seems like the blood samples were taken after PSG. To clarify, were any of the patients on treatment at the time of the blood samples (it was unclear to me when recruitment was)?

Thank you for the question. All patients were recruited prior to initiation of treatment (if deemed necessary by the treating physician).
This statement has been added to the methodology section. “Patients who fulfilled the inclusion criteria were approached, explained and informed regarding the main objectives of the study ***prior to the initiation of mechanical ventilation, if required.”***

Thank you again for your suggestions. We look forward to your positive review.

Sincerely yours,

**Corresponding author:**

**Prof Dr Rohana Abdul Ghani**

Consultant Endocrinologist
Endocrine Unit

Department of Internal Medicine

Faculty of Medicine

Universiti Teknologi MARA (UiTM),

Selangor, Malaysia.
