# Supplementary material for: Significantly higher atherosclerosis risks in patients with obstructive sleep apnea and non-alcoholic fatty liver disease
Source: PLoS One. 2021 Jun 30;16(6):e0253298. doi: 10.1371/journal.pone.0253298 (PMC8244858; doi:10.1371/journal.pone.0253298)
Supplement: Attachment — Submitted filename: Response Letter for Reviewers 3.docx [file pone.0253298.s004.docx]

14^th^ May 2021

Editor

PLOS ONE

**Significantly Higher Atherosclerosis Risks in Patients with Obstructive Sleep Apnoea and Non Alcoholic Fatty Liver Disease** **[PONE-D-21-03573R1]**

Dear Sir,

Thank you again for your kind responses and effort towards this manuscript. I hereby provide the details of the amendments as required by Reviewer 2.

Reviewer #2: Thank you for the responses- a minor point. "Patients who fulfilled
the inclusion criteria were approached, explained and informed regarding the main objectives
of the study prior to the initiation of mechanical ventilation, if required." Can you change mechanical ventilation to "positive airway pressure". Can you also explicitly state that samples were taken prior to initiation of therapy (this only says they were recruited prior to treatment).

This sentence has been further amended to: “Patients who fulfilled the inclusion criteria were approached, explained and informed regarding the main objectives of the study and blood samples were subsequently taken, prior to the initiation of positive airway pressure, if required.”

Thank you again for your suggestions. We look forward to your positive review.

Sincerely yours,

**Corresponding author:**

**Prof Dr Rohana Abdul Ghani**

Consultant Endocrinologist
Endocrine Unit

Department of Internal Medicine

Faculty of Medicine

Universiti Teknologi MARA (UiTM),

Selangor, Malaysia.
